# EARLY WARNING SYSTEM SCORE FOR URGENT ET TUBE OR UNPLANNED ICU ADMISSION FOR ELDERLY PATIENTS IN GENERAL WARDS

**DOI:** 10.1093/geroni/igad104.2982

**Published:** 2023-12-21

**Authors:** Sunee Suwanpasu, Sukanya Poonsap, Werapattra Praparpak

**Affiliations:** KCMH, Sao Ching Cha , Krung Thep, Thailand; King Chulalongkorn Memorial Hospital, Pathumwan, Krung Thep, Thailand; King Chulalongkorn Memorial Hospital, Pathumwan, Krung Thep, Thailand

## Abstract

Early warning system scores are tools used to identify the early signs of physiologic deterioration in order to initiate early intervention and management. This study aims to investigate the efficacy of the National Early Warning Score (NEWS) score and Search out Severity Score (SOS) in predicting urgent ET tube or unplanned ICU admission. A Prospective analytical study of 85 patients aged 60 years and over in general medical wards, was conducted to assess the ability of SOS and NEWS. Prediction ability was assessed using sensitivity, specificity, accuracy, and area under the receiver operating characteristic curve (AUC). The NEWS and SOS systems at 4 hours before the event were the most effective, with the cut-off values of NEWS> 7 and SOS> 4 giving the best accuracy (sensitivity 71.4%, 64.3% specificity 98.2%, 98.2%. Respectively). The area under the ROC curve [AUC]) was 0.90 (95% CI 0.81-0.99) and 0.86 (95% CI 0.75-0.97), respectively. The prediction efficiency decreased over time before the event. However, at 8, 12, and 24 hours before the incident, it was still accurate at a good-fair level (AUC 0.82, 0.82, 0.74, and 0.77, 0.79, 0.72, respectively) The NEWS system provides excellent accuracy, while the SOS system gives a good accuracy level. Therefore, the NEWS system should be used for the early detection of abnormalities and as a guideline for the rapid response to changes in the vulnerable elderly. It keeps patients out of the crisis and is safe and able to predict worsening patients.

